# Supplementary material for: OpenGenomeBrowser: a versatile, dataset-independent and scalable web platform for genome data management and comparative genomics
Source: BMC Genomics. 2022 Dec 27;23:855. doi: 10.1186/s12864-022-09086-3 (PMC9795662; doi:10.1186/s12864-022-09086-3)
Supplement: Supplementary file 1 — Additional file 1: Table S1. Comparison of OpenGenomeBrowser’s features with alternative software platforms. Legend: ✔: feature present; ¢: feature present, but with limitations; Ñ: feature absent. Features were inferred to the best of our knowledge. [file 12864_2022_9086_MOESM1_ESM.pdf]

	OpenGenomeBrowser	ChlamDB	Genomicus	MicroScope	CoGe	MicrobesOnline	WormBase
**Taxonomic range**	Microbes, simple genomes like S. cerevisiae	Chlamydiae	Eukaryotes	Microbes	Entire tree of life	Microbes	Nematodes
**First released**	2022	2019	2010	2009	2008	2003	1996
**Active development**	✓	✓	✓	✓	✓	✗	✓
**Web framework**	Django	Django	Pearl CGI	PHP 5	Pearl CGI	Pearl CGI	Pearl CGI / Lisp
**Source code available**	✓	✓	○ on request	✗ proprietary	✓	✓	✓
**Self-hostable**	✓ easy, full stack using docker compose	○ very difficult	?	✗	○ very difficult	○ very difficult	○ partly dockerized very difficult
**Genome metadata curation through interface**	✓ TaxId, metadata, tags	✗	✗	✗	✗	✗	✗
**Custom annotation types**	✓	✗	✗	✗ however: many annotation sources	✗	✗	✗
**BLAST**	✓	✓	✓	✓	✓	✓	✓
**Phylogenetic trees**	✓	✓	✓	✓	✓	✓	✗
**Gene alignment**	✓	✗	✗	✓ external viewer	✓	✓ external viewer	✓
**Annotation search**	✓	✓	○	○	○	✓	○
**Pathways**	✓ many genomes, groups of genomes	○ limited to one genome via KEGG site	✗	○ MicroCyc, KEGG; limited to one genome via KEGG site	✗	○ limited to two genomes	✗
**Dot plot**	✓	✗	✓ PhylDiagView	○ similar feature (lineplot)	○ no annotations (SynMap2)	✗	○
**Gene loci comparison**	✓	✓	✓	○	✓ GeCoViz	○	○
**Classical genome browser e.g. IGV or Jbrowse**	✗	✗	✗	✓	✓	✗	✓
**Gene trait matching**	✓	✗	✗	○ similar feature (phyloprofile)	✗	✗	✗
