# Supplementary material for: OpenGenomeBrowser: a versatile, dataset-independent and scalable web platform for genome data management and comparative genomics
Source: BMC Genomics. 2022 Dec 27;23:855. doi: 10.1186/s12864-022-09086-3 (PMC9795662; doi:10.1186/s12864-022-09086-3)
Supplement: Supplementary file 2 — Additional file 2: Fig. S1. Detail views. (A) Genome detail view: Shows genome-associated metadata. (B) Gene detail view: Displays a gene’s annotations, nucleotide- and protein sequence, metadata extracted from the GenBank file, as well as an interactive plot that shows the adjacent genes. (C) Tag detail view: Shows the tag’s name, its description and the organisms and genomes that have it. (D) TaxId detail view: Shows the NCBI TaxId, its taxonomic rank, its parent TaxId and the organisms and their genomes that belong to it. [file 12864_2022_9086_MOESM2_ESM.pdf]

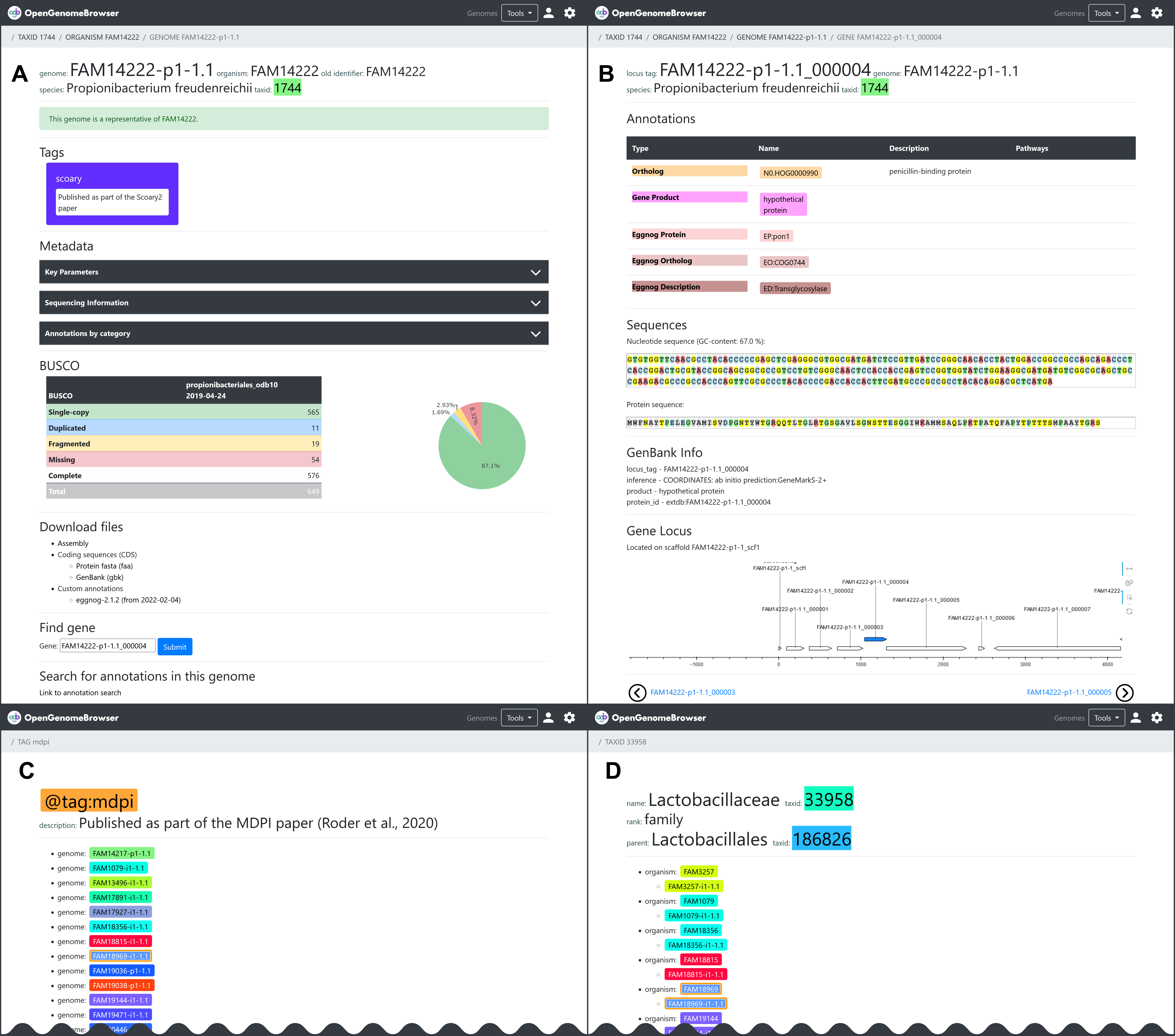
OpenGenomeBrowser
Genomes
Tools
/ TAXID 1744 / ORGANISM FAM14222 / GENOME FAM14222-p1-1.1
A
genome: FAM14222-p1-1.1 organism: FAM14222 old identifier: FAM14222
species: Propionibacterium freudenreichii taxid: 1744
This genome is a representative of FAM14222.
Tags
scoary
Published as part of the Scoary2 paper
Metadata
Key Parameters
Sequencing Information
Annotations by category
BUSCO
BUSCO propionibacteriales_odb10 2019-04-24
Single-copy 565
Duplicated 11
Fragmented 19
Missing 54
Complete 576
Total 649
2.93%
1.69%
8.32%
87.1%
Download files
Assembly
Coding sequences (CDS)
Protein fasta (faa)
GenBank (gbk)
Custom annotations
eggnog-2.1.2 (from 2022-02-04)
Find gene
Gene: FAM14222-p1-1.1_000004
Submit
Search for annotations in this genome
Link to annotation search
/ TAXID 1744 / ORGANISM FAM14222 / GENOME FAM14222-p1-1.1 / GENE FAM14222-p1-1.1_000004
B
locus tag: FAM14222-p1-1.1_000004 genome: FAM14222-p1-1.1
species: Propionibacterium freudenreichii taxid: 1744
Annotations
Type Name Description Pathways
Ortholog N0.HOG0000990 penicillin-binding protein
Gene Product hypothetical protein
Eggnog Protein EP:pon1
Eggnog Ortholog EO:COG0744
Eggnog Description ED:Transglycosylase
Sequences
Nucleotide sequence (GC-content: 67.0 %):
Protein sequence:
GenBank Info
locus_tag - FAM14222-p1-1.1_000004
inference - COORDINATES: ab initio prediction:GeneMarkS-2+
product - hypothetical protein
protein_id - extdb:FAM14222-p1-1.1_000004
Gene Locus
Located on scaffold FAM14222-p1-1_scf1
FAM14222-p1-1_scf1
FAM14222-p1-1.1_000001
FAM14222-p1-1.1_000002
FAM14222-p1-1.1_000003
FAM14222-p1-1.1_000004
FAM14222-p1-1.1_000005
FAM14222-p1-1.1_000006
FAM14222-p1-1.1_000007
−1000
0
1000
2000
3000
4000
FAM14222-p1-1.1_000003
FAM14222-p1-1.1_000005
/ TAG mdpi
C
@tag:mdpi
description: Published as part of the MDPI paper (Roder et al., 2020)
genome: FAM14217-p1-1.1
genome: FAM1079-i1-1.1
genome: FAM13496-i1-1.1
genome: FAM17891-i1-1.1
genome: FAM17927-i1-1.1
genome: FAM18356-i1-1.1
genome: FAM18815-i1-1.1
genome: FAM18969-i1-1.1
genome: FAM19036-p1-1.1
genome: FAM19038-p1-1.1
genome: FAM19144-i1-1.1
genome: FAM19471-i1-1.1
/ TAXID 33958
D
name: Lactobacillaceae taxid: 33958
rank: family
parent: Lactobacillales taxid: 186826
organism: FAM3257
FAM3257-i1-1.1
organism: FAM1079
FAM1079-i1-1.1
organism: FAM18356
FAM18356-i1-1.1
organism: FAM18815
FAM18815-i1-1.1
organism: FAM18969
FAM18969-i1-1.1
organism: FAM19144
